# Dr. E. M. Hale’s Cactaceæ

**Published:** 1889-12

**Authors:** E. M. Hale

**Affiliations:** Chicago, Ill., No. 65 Twenty-second Street


### DR. E. M. HALE'S CACTACEÆ.

As a member of the Bureau of Materia Medica and Therapeutics in the American Institute of Homœopathy, I have selected as the subject of my paper, "The Pathogenetic and Therapeutic properties of the *Cactaceæ*."

The number of known *genera* in this *family* is eighteen, and of *species* about eight hundred. I desire to include in my paper all medical information concerning any species. I urgently solicit physicians of any country to send me all observations relating to the toxic and curative powers of any member of this important family before June 1st, 1890.

E. M. HALE, M. D.

CHICAGO, ILL., No. 65 Twenty-second Street.

